# The Impact of the Virtual Cognitive Health Program on the Cognition and Mental Health of Older Adults: Pre-Post 12-Month Pilot Study

**DOI:** 10.2196/12031

**Published:** 2018-11-09

**Authors:** Shefali Kumar, Jennifer LA Tran, Heidi Moseson, Caroline Tai, Jordan M Glenn, Erica N Madero, Caitlyn Krebs, Nicholas Bott, Jessie L Juusola

**Affiliations:** 1 Evidation Health San Mateo, CA United States; 2 Neurotrack Technologies, Inc Redwood City, CA United States; 3 Department of Medicine School of Medicine Stanford University Stanford, CA United States; 4 PGSP-Stanford PsyD Consortium Department of Clinical Psychology Palo Alto University Palo Alto, CA United States

**Keywords:** cognitive impairment, dementia, Alzheimer disease, mental health

## Abstract

**Background:**

Face-to-face multidomain lifestyle interventions have shown to be effective for improving or maintaining cognitive function in older adults at risk for dementia. Remotely delivered interventions could increase access to such solutions but first require evidence to support that these programs can successfully impact health outcomes.

**Objective:**

The objective of this study was to evaluate the impact of a remotely delivered multidomain lifestyle intervention, the virtual cognitive health (VC Health) program, on the cognitive function and mental health of older adults with subjective cognitive decline (SCD).

**Methods:**

A 52-week, prospective, single-arm, pre-post, remote nationwide clinical trial was conducted to measure the change in cognitive function, depression, and anxiety levels for older adults at risk of developing dementia who participated in the VC Health program. A Web-based study platform was used to screen, consent, and enroll participants across the United States. Participants completed the Repeatable Battery for the Assessment of Neuropsychological Status (RBANS) test and Web-based assessments (which included the Patient Health Questionnaire [PHQ-9] and Generalized Anxiety Disorder [GAD-7] surveys) at baseline and weeks 12, 24, and 52; all data were collected remotely. Changes in RBANS, PHQ-9, and GAD-7 were assessed using 2-tailed paired *t* tests and nonparametric signed-rank tests.

**Results:**

Participants (N=82) were, on average, aged 64 years (range 60.0-74.9 years), 74% (61/82) female, 88% (72/82) white, and 67% (55/82) had a college degree or higher. At baseline, participants had a mean and median RBANS Total Index score of 95.9 (SD 11.1) and 95.5 (interquartile range, IQR=13). Participants experienced a mean and median increase of 5.8 (SD 7.4) and 6 (IQR=11) in RBANS Total Index score from baseline to week 52 (*P*<.001). Participants had a mean and median PHQ-9 score of 8.5 (SD 4.9) and 8 (IQR=6) at baseline and experienced a mean and median decrease of 3.8 (SD 4.1) and 4 (IQR=6) units in PHQ-9 score from baseline to week 52 (*P*<.001). At baseline, participants had a mean and median GAD-7 score of 6.2 (SD 4.5) and 5.5 (IQR=6) and experienced a mean and median decrease of 2.9 (SD 4.1) and 2 (IQR=5) units in GAD-7 score from baseline to week 52 (*P*<.001). Participants were engaged and very satisfied with various program components.

**Conclusions:**

In this study, older adults with SCD who were at risk for dementia experienced statistically significant improvements in their cognitive function, depression, and anxiety levels. These findings serve as initial evidence for the overall feasibility and effectiveness of the VC Health program to improve or maintain cognitive function in older adults who are experiencing SCD. Further research should be conducted to understand the degree to which the improvements are attributable to specific components of the intervention.

**Trial Registration:**

ClinicalTrials.gov NCT02969460; https://clinicaltrials.gov/ct2/show/NCT02969460 (Archived by WebCite at http://www.webcitation.org/73XOph9Qm)

## Introduction

### Background

By 2035, the number of individuals older than 65 years in the United States is projected to outnumber those younger than 18 years [1]. With age, many individuals begin to experience cognitive changes that affect memory, learning, language, and judgment, all of which can impact the ability to perform daily tasks [2]. In the early stages of cognitive change, individuals may experience subjective cognitive decline (SCD), in which subjective changes in memory and cognition are perceived but are not associated with clinically measurable abnormalities [3,4]. These individuals are considered at-risk for developing dementia, particularly Alzheimer disease (AD) [5-7]. If cognitive decline continues to worsen, SCD can progress to mild cognitive impairment, an intermediate state between normal cognition and dementia, in which there are clinically observable changes in measures of cognitive function [4-8].

Dementia places a significant burden on the health care system in terms of cost and caregiver hours. With a lifetime cost of care estimated at US $341,000 per individual, dementia is considered to be one of the world’s costliest health conditions [9]. Total health care expenditures for AD and other dementias in 2018 was estimated at US $277 billion, and this cost is expected to be more than US $1 trillion by 2050 [9]. In addition, family and other unpaid caregivers provided an estimated 18.4 billion hours of care (valued at over US $232 billion) to individuals with dementia [9]. Given the high burden of dementia and current lack of efficacious pharmaceutical agents, there is significant value in developing early interventions to help prevent or delay the onset and progression of disease.

Multidomain interventions that target modifiable lifestyle-related risk factors (eg, nutritional intake, physical activity, and cognitive engagement) can be useful prevention tools, as many studies have linked vascular and lifestyle-related risk factors to an increased risk of cognitive impairment and dementia [10-12]. The Finnish Geriatric Intervention Study to Prevent Cognitive Impairment and Disability (FINGER) found that a 2-year multidomain intervention of diet, exercise, vascular risk monitoring, and cognitive training could help at-risk older individuals improve or maintain their cognitive functioning [13,14]. Following the success of the FINGER study, numerous replication studies have been launched globally, including the Singapore Intervention Study to Prevent Cognitive Impairment and Disability, Multimodal Intervention to Delay Dementia and Disability in Rural China, and United States Study to Protect Brain Health Through Lifestyle Intervention to Reduce Risk studies [15-17]. However, many challenges related to access, cost, and other logistical constraints remain for these multidomain programs. In the current format, individuals are required to visit an office for in-person coaching and assessment, limiting the scalability and reach of the intervention. As new technological developments change the way health care services are delivered, digitally administered lifestyle programs can be an effective and efficient way to help older individuals at risk for developing dementia improve or maintain their cognitive function [18].

### The Virtual Cognitive Health Program

The Virtual Cognitive (VC) Health program, which was modeled after the FINGER study, is a commercially available, multidomain digital lifestyle intervention for the prevention or delay of cognitive impairment in at-risk aging adults. The year-long digital intervention includes exercise and nutritional guidance, cognitive training, social engagement, and personalized health coaching. The VC Health study aimed to understand the feasibility and effectiveness of the VC Health program, specifically examining the long-term impact this program may have on cognitive function and mental health.

## Methods

### Study Overview

The VC Health study was a 52-week, prospective, intention-to-treat (ITT), single-arm, pre-post, remote nationwide clinical trial that aimed to evaluate change in cognitive function and mental health of individuals at-risk for dementia with SCD. A Web-based study platform (Achievement Studies, Evidation Health; San Mateo, CA) was used to screen, consent, and enroll study participants across the United States. The platform was also used to collect study data and monitor completion of study tasks. Participants were able to complete all study procedures from their own homes using a computer with internet access and a webcam. The full details of the study design have been published previously [19], and the study was registered on ClinicalTrials.gov (NCT02969460). The trial was reviewed and approved by the Solutions institutional review board (Little Rock, AR).

### Recruitment and Screening

Various digital platforms (eg, online patient communities, social media, and targeted advertisements) were utilized to recruit study participants. All interested candidates were asked to complete a Web-based screener via the study platform to assess eligibility. Eligible individuals were aged between 60 and 75 years and showed signs of SCD as assessed by scoring ≥1 on the Subjective Cognitive Decline Questionnaire (SCD-9) [20] and endorsing the 1-item subjective cognitive decline with worry item (“Do you feel like your memory is becoming worse?” Possible responses were “No,” “Yes, but this does not worry me,” or “Yes, this worries me”) [21]. Individuals also needed to have the ability to make and receive phone calls and text messages; have access to a desktop computer with video teleconferencing and a reliable internet connection, which were required to access the intervention and complete the study procedures; and be motivated to use a daily coaching program, as assessed by self-reported willingness (on a 5-point scale from extremely willing to not at all willing) to participate in the virtual cognitive training coaching program. Individuals were excluded if they had a significant history of dementia, mental illness, substance abuse, learning disability, or neurologic conditions, had ophthalmologic or visual problems (eg, legal blindness, detached retinas, and occlusive cataracts) that prevented them from viewing a computer screen at a normal distance, were currently participating in a formal cognitive-training coaching program, or were currently pregnant.

### Enrollment and Study Procedures

All eligible participants completed an electronic informed consent form through the Web-based study platform and were asked to complete a Web-based baseline assessment that included questions about demographics, average nightly sleep hours, and weekly diet and exercise habits. Depression and anxiety severity scores from the Patient Health Questionnaire (PHQ-9) and Generalized Anxiety Disorder (GAD-7) survey were also assessed at baseline [22-24]. Upon completion of the baseline assessment, participants were scheduled to complete a 30-min baseline Repeatable Battery for the Assessment of Neuropsychological Status (RBANS) test [25]. A licensed psychologist used video teleconferencing to administer the RBANS test to qualified candidates (the Echelon Group; Woodstock, GA). The validity of digitally administered neuropsychological tests, including RBANS, has been supported by multiple studies [25-28]. After completing the RBANS test, participants began an initial VC Health coaching session over the phone with a program coach. Individuals were considered enrolled in the VC Health study once this initial coaching session was complete.

Throughout the study, the participants were asked to complete follow-up RBANS tests and Web-based assessments at weeks 12, 24, and 52. To reduce practice effects due to repeated testing over time, alternate forms of the RBANS test were used at each time point. The Web-based assessments included questions about sleep, activity levels, depression and anxiety symptom severity (PHQ-9 and GAD-7, respectively), and experience with the VC Health program. Participants also completed coaching sessions focused on nutrition, physical activity, and cognitive training as part of the VC Health program during the first 6 months of the study.

### Intervention: Virtual Cognitive Health Program

The VC Health program is a multidomain lifestyle intervention designed to prevent or delay cognitive decline and impairment in older at-risk adults and comprises 2 phases. The first 6 months of the program (active phase) emphasize lifestyle change, whereas the last 6 months of the program (maintenance phase) emphasize habit reinforcement. The program consists of individually tailored coaching sessions focused on nutrition, physical exercise, and cognitive training. The cognitive training program (provided by MindAgilis; London, England) focused on processing speed, executive function, working memory, episodic memory, and mental speed, which are tasks shown to be associated with improved cognitive ability and less difficulty with instrumental activities of daily living [29,30].

All program coaches were certified as personal trainers with mastery of exercise physiology safety and nutritional health practices. Coaches were available via telephone, email, and text messaging. To promote social engagement, participants were encouraged to participate in an internal social network with communal support and directed life review prompts adapted from life review protocols moderated by a licensed clinical psychologist.

### Study Outcomes

The primary outcome of the VC Health study was change in RBANS Total Index score from baseline to week 24 and week 52. The RBANS test has demonstrated strong efficacy as a dementia assessment tool and can also detect cognitive impairment associated with AD [31-33]. Secondary outcomes included change in PHQ-9 depression severity scores and change in GAD-7 anxiety severity scores from baseline to week 24 and week 52, as both depression and anxiety are risk factors for AD [34,35]. RBANS, PHQ-9, and GAD-7 data were also collected at week 12 to allow for interim nonprimary analysis. Exploratory analyses assessed the association between various engagement measures with the VC Health program and change in RBANS, PHQ-9, and GAD-7 scores.

#### Sample Size and Statistical Analyses

Due to its preliminary nature, the VC Health study was not powered to detect any specific difference in RBANS score. On the basis of the overall capacity of the VC Health program, 85 participants were enrolled in the study.

Baseline sociodemographic characteristics were collected for all individuals in the ITT population. For the primary analysis, mean and median change in RBANS Total Index score from baseline to week 24 and baseline to week 52 were compared using a 2-tailed paired *t* test and a nonparametric signed-rank test. Similar analyses were conducted to evaluate the mean and median changes in RBANS Total Index score and the 5 RBANS Sub-Index scores (immediate memory, visuospatial, language, attention, and delayed memory) across various time points (baseline to week 24, baseline to week 52, and week 24 to week 52).

For the secondary analysis, mean and median change in PHQ-9 and GAD-7 from baseline to week 24 and baseline to week 52 were assessed using a 2-tailed paired *t* test and a nonparametric signed-rank test. Similar analyses were conducted to measure the mean and median changes in PHQ-9 and GAD-7 across additional time points (baseline to week 24, baseline to week 52, and week 24 to week 52).

We also conducted an unadjusted repeated measures analysis using a linear mixed model with a random intercept specific for each participant to account for intraparticipant correlation. The model used baseline; weeks 12, 24, and 52 RBANS; PHQ-9; and GAD-7 scores for the repeated measures analysis. This analysis included all individuals in the analysis set and was not limited to those individuals with complete data at baseline and week 52. Results for RBANS Total Index score, PHQ-9, and GAD-7 were stratified by key participant characteristics, including sex (female vs male), ethnicity (white vs nonwhite), and education level (college degree or higher vs less than college degree). Supplemental analyses used a linear regression model to explore the relationship between various user engagement metrics such as number of coaching calls and changes in RBANS, PHQ-9, and GAD-7 scores. All analyses were conducted in Stata version 14.2 or R version 3.5.0, with an alpha of .05 for assessment of statistical significance.

## Results

### Study Sample

**Figure 1:**
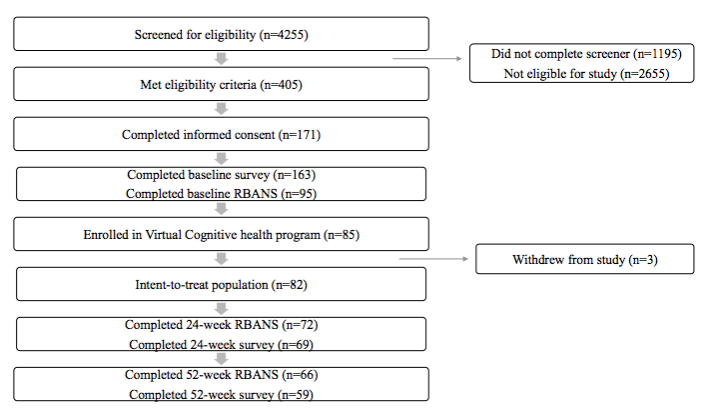
Study diagram. RBANS: Repeatable Battery for the Assessment of Neuropsychological Status.

Of the 4255 individuals who initiated the Web-based screening process, 28.08% (1195/4255) did not complete the screener, 62.40% (2655/4255) completed the screener but were deemed ineligible for the study, and 9.52% (405/4255) completed the screener and were eligible for the study. Among all 3060 individuals who completed the screener, the top reasons for study ineligibility included not endorsing the 1-item subjective cognitive decline with worry question (80.03%, 2449/3060), not willing to use a virtual coaching program (14.28%, 437/3060), and an SCD-9 score <1 (11.96%, 366/3060). Of the 405 individuals who completed the screener, 57.8% (234/405) did not complete the informed consent process. Of the 171 individuals who completed informed consent, the majority (95.3%, 163/171) completed the baseline survey. A total of 68 individuals (41.7%, 68/163) of the 163 participants who completed the baseline survey did not schedule or complete the initial RBANS test. Of the 95 individuals who completed the initial RBANS test, 11% (10/95) never scheduled or completed their initial VC Health program coaching session. A total of 85 individuals completed all of the required steps, including the initial coaching session, and enrolled in the study. A total of 3 participants withdrew from the study because they no longer wanted to participate in the program or study. As such, 82 participants were included in the analysis set (Figure 1).

Table 1 details the characteristics of the study population. A majority of the study population was female (74%, 61/82), white (88%, 72/82), and had a college degree or higher (67%, 55/82). Mean age of the population was 64 years (range 60-74.9 years). The ITT population represented a geographically diverse population comprising 29 different states in the United States [19].

### Primary Outcome: Cognition

At the 24-week time point, RBANS Total Index score decreased from baseline, but the change was not statistically significant (*P*=.15). Among the 72 participants who completed both their baseline and week 24 RBANS test, mean and median RBANS Total Index score was 96.3 (SD 11.2) and 96 (IQR=13) at baseline, and 95.0 (SD 9.3) and 95 (IQR=13) at week 24, respectively, for an unadjusted mean and median change of −1.4 (SD 8.1) and 0 (IQR=10) units (Table 2). Of the 5 Sub-Index scores, participants experienced a statistically significant change (a slight decrease) in the immediate memory domain (*P=*.02; Table 2).

**Table 1:** Baseline characteristics of study population (N=82).

Characteristics at baseline		Statistics
Age in years, mean (SD)		64 (4)
**Gender, n (%)**		
	Male	20 (24)
	Female	61 (74)
	Other	1 (1)
**Education, n (%)**		
	High school graduate or general education development degree	3 (4)
	Trade, technical or vocational training	2 (2)
	Some college, no degree	22 (27)
	College graduate, associate’s or bachelor’s	29 (35)
	Graduate degree	19 (23)
	Doctorate degree	7 (9)
**Race/ethnicity, n (%)**		
	African American	5 (6)
	Asian	1 (1)
	White	72 (88)
	Hispanic	3 (4)
	Other	1 (1)
**BMI^a^, n (%)**		
	Underweight, <18.5 kg/m^2^	0 (0)
	Healthy weight, 18.5-24.9 kg/m^2^	16 (20)
	Overweight, 25.0-29.9 kg/m^2^	24 (29)
	Obese, 30-34.9 kg/m^2^	25 (30)
	Very obese, ≥35 kg/m^2^	15 (18)
**Average nightly sleep duration, n (%)**		
	<3 hours	1 (1)
	4 to 5 hours	4 (5)
	5 to 6 hours	18 (22)
	6 to 7 hours	35 (43)
	7 to 8 hours	16 (20)
	8 to 9 hours	6 (7)
	9 to 10 hours	0 (0)
	>10 hours	2 (2)

^a^BMI: body mass index.

At the 52-week time point, participants experienced a statistically significant increase in RBANS Total Index score from baseline (*P*<.001). Among the 66 participants who completed both their baseline and week 52 RBANS test, mean and median RBANS Total Index score was 96.9 (SD 10.7) and 97 (IQR=15) at baseline, and 102.7 (SD 9.9) and 101.5 (IQR=13) at week 52, respectively, for an unadjusted mean and median increase of 5.8 (SD 7.4) and 6 (IQR=11) units (*P*<.001; Table 2). Of the 5 Sub-Index scores, participants experienced a statistically significant increase from baseline to week 52 in immediate memory, language, and delayed memory Sub-Index scores (*P*<.001; Table 2).

**Figure 2:**
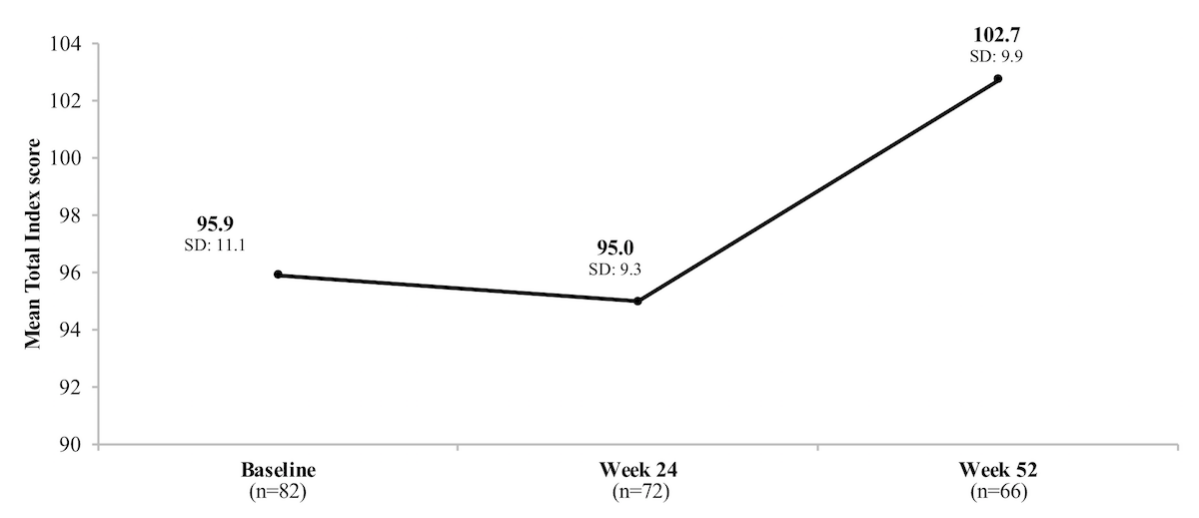
Change in mean Repeatable Battery for the Assessment of Neuropsychological Status (RBANS) Total Index score over time.

Comprehensive mean and median RBANS Total Index scores for all individuals who completed the RBANS test at each study time point are shown in Table 3 and Figure 2.

**Table 2:** Change in mean and median Repeatable Battery for the Assessment of Neuropsychological Status (RBANS) scores.

Metric			N	Mean (SD)^a^	Median (IQR^b^)^c^
**Total Index score**					
		Baseline to week 24 change	72	−1.4 (8.1)	0 (10)
		Baseline to week 52 change	66	5.8 (7.4)^d^	6 (11)^d^
		Week 24 to week 52 change	64	6.8 (6.3)^d^	6 (8)^d^
**Immediate memory**					
		Baseline to week 24 change	72	−3.7 (12.7)^f^	−3.5 (14.5)^e^
		Baseline to week 52 change	66	7.8 (11.4)^d^	8 (15)^d^
		Week 24 to week 52 change	64	12.2 (11.3)^d^	12 (17)^d^
**Visuospatial**					
		Baseline to week 24 change	72	−0.5 (14.9)	0 (21.5)
		Baseline to week 52 change	66	0.5 (13.7)	0 (22)
		Week 24 to week 52 change	64	0.3 (11.1)	0 (16)
**Language**					
		Baseline to week 24 change	72	0.9 (11.0)	2 (11.5)
		Baseline to week 52 change	66	5.7 (12.5)^d^	5.5 (15)^d^
		Week 24 to week 52 change	64	4.5 (9.7)^d^	5 (11.5)^d^
**Attention**					
		Baseline to week 24 change	72	−1.5 (12.1)	−3 (13)
		Baseline to week 52 change	66	2.0 (12.6)	0 (16)
		Week 24 to week 52 change	64	3.5 (9.7)^e^	3 (13)^e^
**Delayed memory**					
	Baseline to week 24 change		72	0.9 (10.5)	0 (9.5)
	Baseline to week 52 change		66	5.4 (12.5)^d^	4 (14)^d^
	Week 24 to week 52 change		64	3.6 (9.0)^e^	4 (7)^d^

^a^*P* values in the mean column are calculated using 2-sided paired *t* tests to compare means at the 2 time points.

^b^IQR: interquartile range.

^c^*P* values in the median column are calculated using 2-sided nonparametric signed-rank tests to compare distributions at the 2 time points.

^d^*P*<.001.

^e^*P*<.01.

^f^*P*=.02.

### Secondary Outcomes: Mental Health

Depression symptom levels (as measured by PHQ-9) decreased from baseline to week 24. Among the 69 participants who completed both their baseline and week 24 PHQ-9 tests, mean and median PHQ-9 was 8.6 (SD 4.8) and 8 (IQR=6) at baseline and 5.5 (SD 5.2) and 4 (IQR=6) at week 24, respectively, for a statistically significant unadjusted mean and median PHQ-9 change of −3.1 (SD 4.7) and −3 (IQR=6) units (*P*<.001; Table 4). At the 52-week time point, depression symptom levels also decreased from baseline. Among the 59 participants who completed both their baseline and week 52 PHQ-9 test, mean and median PHQ-9 were 8.3 (SD 5.0) and 8.0 (IQR=6) at baseline and 4.5 (SD 4.5) and 3.0 (IQR=5) at week 52, respectively, for a statistically significant unadjusted mean and median PHQ-9 change of −3.8 (SD 4.1) and −4 (IQR=6) units (*P*<.001; Table 4).

Similar results were observed with anxiety symptom levels (as measured by GAD-7). Among the 69 participants who completed both their baseline and week 24 GAD-7 tests, mean and median GAD-7 were 6.2 (SD 4.4) and 6 (IQR=6) at baseline and 3.9 (SD 4.6) and 3 (IQR=4) at week 24, respectively, for a statistically significant unadjusted mean and median GAD-7 change of −2.3 (SD 4.3) and −2 (IQR=4) units (*P*<.001; Table 4). Among the 59 participants who completed both their baseline and week 52 GAD-7 test, mean and median GAD-7 were 6.0 (SD 4.7) and 5 (IQR=7) at baseline and 3.1 (SD 4.1) and 2 (IQR=4) at week 52, respectively, for a statistically significant unadjusted mean and median GAD-7 change of −2.9 (SD 4.1) and −2 (IQR=5) units (*P*<.001; Table 4).

**Figure 3:**
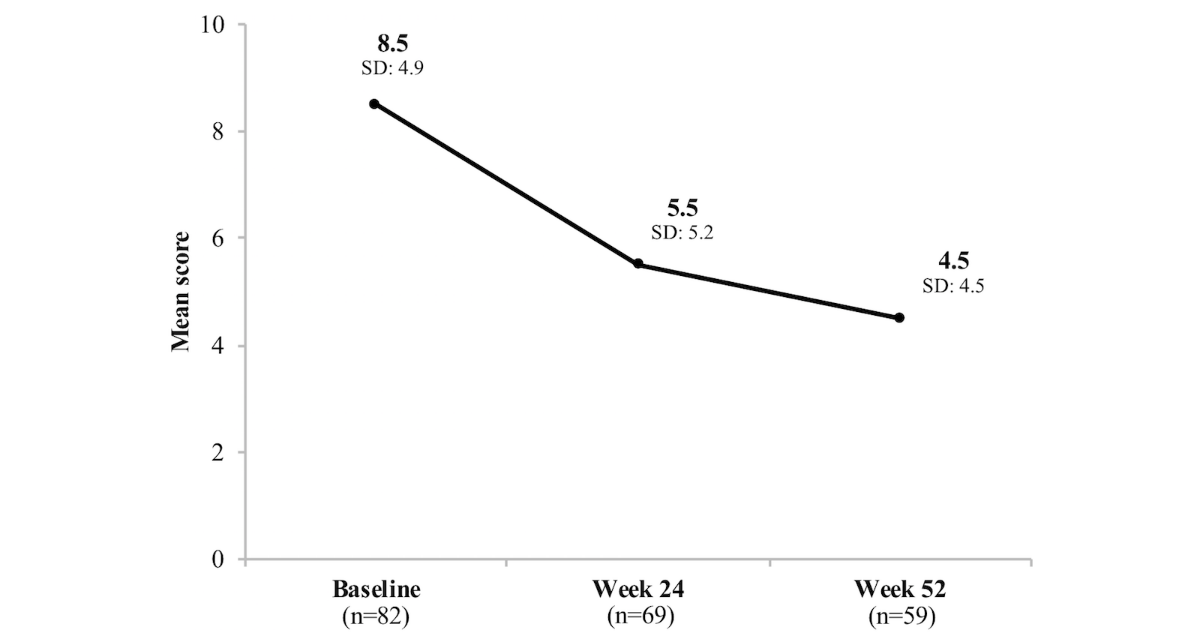
Change in Patient Health Questionnaire-9 (PHQ-9) scores over time.

**Figure 4:**
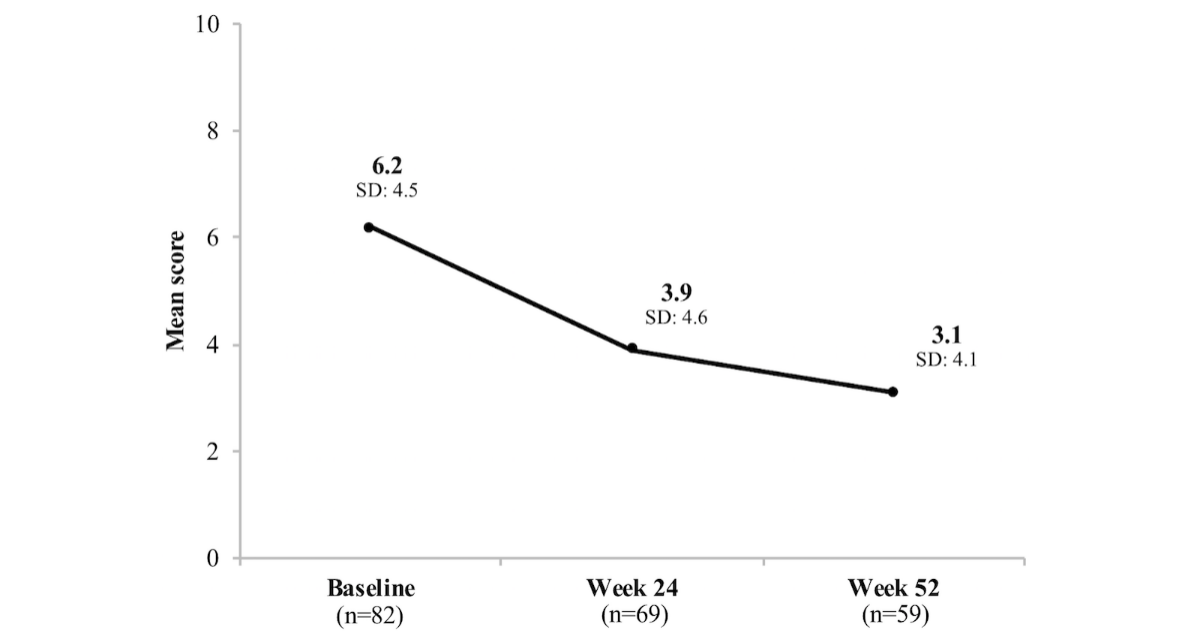
Change in Generalized Anxiety Disorder-7 (GAD-7) scores over time.

Comprehensive mean and median PHQ-9 and GAD-7 scores for all individuals who completed the assessments at each study time point are shown in Table 5 and Figures 3 and 4.

### Primary and Secondary Outcomes: Mixed-Effects Model Analysis

In the repeated measures analysis using a mixed-effects model with a random intercept specific for each participant in the study population (N=82), we found that the marginal mean change in RBANS Total Index score was consistent with our results from the primary analysis. Using a mixed-effects model, we found that the marginal mean change in RBANS Total Index score was −1.3 (95% CI −3.0 to 0.4) from baseline to week 24 (*P*=.14) and 5.7 (95% CI 3.9 to 7.5) from baseline to week 52 (*P*<.001).

**Table 3:** Mean and median Repeatable Battery for the Assessment of Neuropsychological Status (RBANS) scores.

Metric			N		Mean (SD)		Median (IQR^a^)
**Total Index score**							
	Baseline	82		95.9 (11.1)		95.5 (13)	
	Week 12	73		100.8 (11.7)		100 (18)	
	Week 24	72		95.0 (9.3)		95 (13)	
	Week 52	66		102.7 (9.9)		101.5 (13)	
**Immediate memory**							
	Baseline	82		99.4 (12.5)		103 (22)	
	Week 12	73		100.8 (13.0)		103 (19)	
	Week 24	72		95.4 (13.2)		97 (21)	
	Week 52	66		107.4 (10.8)		109 (11)	
**Visuospatial**							
	Baseline	82		90.4 (14.3)		87 (19)	
	Week 12	73		95.5 (13.2)		96 (16)	
	Week 24	72		90.1 (12.1)		89 (18.5)	
	Week 52	66		91.2 (12.2)		92 (21)	
**Language**							
	Baseline	82		97.2 (10.2)		96 (9)	
	Week 12	73		104 (10.7)		104 (13)	
	Week 24	72		98.9 (8.1)		98 (10.5)	
	Week 52	66		103.8 (9.3)		101 (12)	
**Attention**							
	Baseline	82		102.8 (14.8)		101.5 (24)	
	Week 12	73		103.3 (16.5)		103 (21)	
	Week 24	72		102.1 (12.7)		103 (19.5)	
	Week 52	66		107.1 (14.7)		106 (18)	
**Delayed memory**							
	Baseline	82		96.6 (12.5)		98 (20)	
	Week 12	73		100.8 (13.3)		102 (15)	
	Week 24	72		97.6 (9.4)		98 (7)	
	Week 52	66		102.2 (10.4)		102 (12)	

^a^IQR: interquartile range.

**Table 4:** Change in mean and median Patient Health Questionnaire-9 (PHQ-9) and Generalized Anxiety Disorder-7 survey (GAD-7) scores.

Metric		N	Mean (SD)^a^	Median (IQR^b^)^c^
**PHQ-9 total score**				
	Baseline to week 24 change	69	−3.1 (4.7)^d^	−3 (6)^d^
	Baseline to week 52 change	59	−3.8 (4.1)^d^	−4 (6)^d^
	Week 24 to week 52 change	56	−0.8 (3.6)	−1 (4)^e^
**GAD-7 total score**				
	Baseline to week 24 change	69	−2.3 (4.3)^d^	−2 (4)^d^
	Baseline to week 52 change	59	−2.9 (4.1)^d^	−2 (5)^d^
	Week 24 to week 52 change	56	−0.8 (3.7)	0 (3.5)

^a^*P* values in the mean column are calculated using two-sided paired *t* tests to compare means at the 2 time points.

^b^IQR: interquartile range.

^c^*P* values in the median column are calculated using two-sided nonparametric signed-rank tests to compare distributions at the 2 time points.

^d^*P*<.001.

^e^*P*=.01.

We conducted similar analyses for PHQ-9 and GAD-7 scores. Using a mixed-effects model, we found that the marginal mean change in PHQ-9 score was −3.0 (95% CI −4.1 to −2.0) from baseline to week 24 (*P*<.001) and −3.8 (95% CI −4.8 to −2.9) from baseline to week 52 (*P*<.001). The marginal mean change in GAD-7 score was −2.3 (95% CI −3.3 to −1.4) from baseline to week 24 (*P*<.001) and −3.0 (95% CI −4.0 to −2.1) from baseline to week 52 (*P*<.001). These marginal mean changes in PHQ-9 and GAD-7 from the mixed-effects model were similar to the mean changes from our primary analysis.

### Primary and Secondary Outcomes: Stratified by Key Characteristics

Baseline RBANS, PHQ-9, and GAD-7 scores were similar across all demographic subgroups analyzed (Table 6). Mean change in RBANS Total Index score from baseline to week 24 was similar between each respective cohort (Table 7). The increase in RBANS score between baseline and week 52 was significantly higher for females (mean change 7.2 [SD 6.9]) compared with males (mean change 1.1 [SD 7.0]; Table 7); however, the difference in mean change was not significant when stratified by race or education. Stratification did not show any differences when looking at difference in mean change in PHQ-9 and GAD-7 scores from baseline to week 24 and baseline to week 52.

### Exploratory Analyses: Lifestyle Factors, Program Satisfaction, and Program Engagement

Participants reported high satisfaction rates with the program. Of study participants who completed the study end questionnaire at 52 weeks (n=59), 86% (51/59) reported that the VC Health program was at least moderately helpful in improving their cognitive ability. Almost 69% (41/59) of participants felt the VC Health program was at least moderately helpful in improving their sleep habits. At baseline, participants reported sleeping on average 6.5 hours (SD 1.3) per night, whereas at week 52, participants reported sleeping 6.6 hours per night (SD 1.2; *P*=.50).

**Table 5:** Mean and median Patient Health Questionnaire-9 (PHQ-9) and Generalized Anxiety Disorder-7 survey (GAD-7) scores.

Metric		N	Mean (SD)	Median (IQR^a^)
**PHQ-9 score**				
	Baseline	82	8.5 (4.9)	8 (6)
	Week 12	54	4.8 (4.0)	3.5 (5)
	Week 24	69	5.5 (5.2)	4 (6)
	Week 52	59	4.5 (4.5)	3 (5)
**GAD-7 score**				
	Baseline	82	6.2 (4.5)	5.5 (6)
	Week 12	54	3.0 (3.4)	2 (5)
	Week 24	69	3.9 (4.6)	3 (4)
	Week 52	59	3.1 (4.1)	2 (4)

^a^IQR: interquartile range.

A total of 86% (51/59) and 80% (47/59) of participants reported that the VC Health program was at least moderately helpful in improving their diet and eating habits, and physical activity levels, respectively. At baseline, participants reported exercising an average of 2.8 days per week (SD 2.4), whereas at week 52, participants reported exercising 3.9 days per week (SD 1.8; *P*=.01). A total of 93% (55/59) of study participants reported that the program made at least some impact on their daily food decisions. Average body mass index (BMI; based on self-reported height and weight) also decreased in the 52-week study period, but this change was not statistically significant; at baseline, BMI was 29.9 kg/m^2^ (SD 6.3), and at the end of the study, BMI was 29.2 kg/m^2^ (SD 5.7; *P*=.07). A majority of participants (75%, 44/59) reported that the VC Health program at least moderately improved their stress levels.

**Table 6:** Change in mean Repeatable Battery for the Assessment of Neuropsychological Status (RBANS), Patient Health Questionnaire-9 (PHQ-9), and Generalized Anxiety Disorder-7 survey (GAD-7) scores stratified by sex, race, and education level.

Outcome		Stratification by sex^a^						Stratification by race							Stratification by education level					
		Female		Male			White				Nonwhite			College degree or above				Below college degree		
		N	Mean change (SD)		N	Mean change (SD)			N	Mean change (SD)		N	Mean change (SD)			N	Mean change (SD)		N	Mean change (SD)
**Total Index score**																				
	Baseline to week 24 change	53	−0.9 (8.4)		18	−3.1 (7.4)			64	−1.1 (7.8)		8	−3.5 (10.8)			48	−0.6 (7.7)		24	−2.8 (8.8)
	Baseline to week 52 change	50	7.2^b^ (7.1)		15	1.1^b^ (7.0)			59	6.1 (7.0)		7	2.6 (10.7)			45	6.8 (7.0)		21	3.6 (8.0)
**PHQ-9 total score**																				
	Baseline to week 24 change	52	−2.8 (5.0)		16	−4.1 (3.2)			60	−3.2 (4.4)		9	−2.9 (6.3)			46	−3.1 (5.1)		23	−3.1 (3.6)
	Baseline to week 52 change	45	−3.6 (4.3)		13	−4.3 (3.7)			54	−3.9 (4.2)		5	−2.6 (3.6)			42	−4.0 (4.2)		17	−3.2 (4.0)
**GAD-7 total score**																				
	Baseline to week 24 change	52	−2.0 (4.6)		16	−2.9 (2.8)			60	−2.0 (4.3)		9	−4.4 (3.7)			46	−2.5 (4.8)		23	−1.8 (2.8)
	Baseline to week 52 change	45	−2.7 (4.4)		13	−3.5 (3.0)			54	−2.8 (4.1)		5	−4.0 (4.1)			42	−3.0 (4.4)		17	−2.6 (3.5)

^a^One participant identified as *other* sex; thus, we do not report data for the *other* category.

^b^*P*=.005 when comparing the difference between females and males.

**Table 7:** Mean Repeatable Battery for the Assessment of Neuropsychological Status (RBANS), Patient Health Questionnaire-9 (PHQ-9), and Generalized Anxiety Disorder-7 survey (GAD-7) scores stratified by sex, race, and education level.

Outcome		Stratification by sex^a^				Stratification by race					Stratification by education level			
		Female		Male		White		Nonwhite		College degree or above			Below college degree	
		N	Mean (SD)	N	Mean (SD)	N	Mean (SD)	N	Mean (SD)	N		Mean (SD)	N	Mean (SD)
**Total Index score**														
	Baseline	61	96.4 (11.0)	20	93.7 (11.1)	72	95.9 (10.6)	10	96.1 (14.9)	55		96.2 (11.2)	27	95.2 (10.9)
	Week 12	55	102.4 (11.2)	17	95.6 (12.2)	66	101.3 (11.1)	7	96.1 (16.4)	48		101.8 (12.2)	25	99.0 (10.5)
	Week 24	53	95.8 (9.2)	18	91.8 (8.7)	64	95.2 (9.0)	8	93.1 (12.1)	48		96.0 (9.6)	24	92.9 (8.6)
	Week 52	50	103.9 (10.4)	15	98.1 (6.8)	59	102.5 (10.1)	7	104.4 (8.7)	45		103.6 (10.1)	21	100.8 (9.5)
**PHQ-9 total score**														
	Baseline	61	8.4 (5.1)	20	9.0 (4.4)	72	8.8 (5.1)	10	6.6 (3.0)	55		8.4 (5.0)	27	8.8 (4.8)
	Week 12	39	4.5 (3.4)	14	5.6 (5.5)	47	5.1 (4.1)	7	3.0 (2.7)	38		4.5 (3.6)	16	5.5 (5.0)
	Week 24	52	5.9 (5.7)	16	4.3 (3.0)	60	5.8 (4.9)	9	3.6 (6.7)	46		5.3 (5.2)	23	5.9 (5.3)
	Week 52	45	4.7 (4.8)	13	3.8 (3.5)	54	4.8 (4.5)	5	2.2 (3.8)	42		4.4 (3.9)	17	4.9 (5.7)
**GAD-7 total score**														
	Baseline	61	6.0 (4.7)	20	6.7 (3.9)	72	6.2 (4.7)	10	6.3 (2.8)	55		6.2 (4.8)	27	6.1 (4.1)
	Week 12	39	3.1 (3.4)	14	3.0 (3.7)	47	3.2 (3.5)	7	2.0 (2.1)	38		2.7 (3.0)	16	3.9 (4.1)
	Week 24	52	3.9 (4.8)	16	3.8 (3.9)	60	4.2 (4.7)	9	1.7 (2.4)	46		3.5 (4.6)	23	4.6 (4.5)
	Week 52	45	3.0 (4.3)	13	2.9 (3.6)	54	3.2 (4.2)	5	2.0 (2.8)	42		3.0 (4.0)	17	3.3 (4.3)

^a^One participant identified as *other* sex; thus, we do not report data for the *other* category.

Overall, the mean and median numbers of coaching calls completed by study participants over the 12-month study period were 16.5 (SD 9.6) and 16.0 (IQR=12.3), respectively. The mean and median call length was 12.9 min (SD 5.9) and 11.7 min (IQR=6.9), respectively. When comparing program engagement and change in RBANS Total Index score, we found no statistically significant association between change in Total RBANS Index score and number of phone calls, number of interactions with the program’s social media platform, or number of participant food and exercise logs. We found a statistically significant inverse association with the number of completed phone calls and change in PHQ-9 (beta=−.16, *P*=.01) and GAD-7 (beta=−.17, *P*=.008) between baseline and week 52. For each additional call, PHQ-9 scores improved by 0.16 units and GAD-7 scores improved by 0.17 units. Lower PHQ-9 and GAD-7 scores correspond to lower levels of depression and anxiety; therefore, a negative change in average PHQ-9 and GAD-7 score from baseline to week 52 indicates an improvement in mental health status. There was also an inverse association between the number of food and exercise logs entered by the participant and change in PHQ-9 (beta=−.01, *P*=.03) and GAD-7 (beta=−.01, *P*=.002). For each additional day that a participant logged their food or exercise, the average PHQ-9 and GAD-7 scores improved by 0.01 units.

## Discussion

### Principal Findings

This study provides a number of key insights into the feasibility and effectiveness of the VC Health program, a remotely delivered multidomain lifestyle intervention designed for the prevention or delay of cognitive impairment in older adults at risk of developing AD. One of the strengths of this study was the 52-week study duration, which allowed us to examine the long-term impact of the VC Health program. Participants did not experience a statistically significant change in cognitive ability from baseline to week 24, but cognitive function was significantly increased from baseline to week 52. On the basis of RBANS scoring guidelines, participants at baseline had an *Average* RBANS Total Index score (scores between 90 and 109 are considered to be *Average*) [36]. By the end of the study, participants experienced an average increase in RBANS Total Index score of 5.8 units, remaining in the *Average* category. It is possible that the full impact of the VC Health program on cognitive ability may completely manifest in the longer term, over the course of multiple years. Future longitudinal studies should assess whether this increase in cognitive function can be sustained or further improved after 1 year, as well as how cognitive function would be expected to change in the absence of an intervention (in a control group).

Study participants also experienced statistically and clinically significant improvements in symptoms of depression and anxiety, as measured by PHQ-9 and GAD-7 scores. At baseline, participants were considered on average to have *Mild Depression* and *Mild Anxiety* based on PHQ-9 and GAD-7 scoring guidelines [22,24]. By the end of the study, their PHQ-9 and GAD-7 scores decreased on average by 3.8 and 2.9 points, respectively, moving participants into the *Minimal or No Depression* and *Minimal or No Anxiety* categories [22,24]. Participants experienced the largest improvement in PHQ-9 and GAD-7 from baseline to week 24, after which they did not experience any additional statistically significant benefits between week 24 and 52. This suggests that the VC Health program’s impact on mental health is more immediate than its impact on cognitive function. At the beginning of the program, the immediate exposure to psychoeducation, cognitive stimulation, and/or frequent coaching sessions may have had an instant positive impact on a participant’s daily life, resulting in improved mental health. Given that depression and anxiety symptoms are known risk factors for AD, mitigating these symptoms in the first half of the program may play a role in the observed long-term improvements in cognitive function. Future longitudinal studies should be conducted to further understand the longer-term effects of the program on depression and anxiety.

When we examined various components of program engagement, there was no association between the number of coaching calls a participant completed and change in RBANS Total Index score over time, but there was an association between number of calls completed and improvement in both PHQ-9 and GAD-7 scores. Coaching calls are only 1 component of the overall program experience, and different participants may require various levels of attention and frequency of interactions with their coach to gain the same benefit in cognitive function from the program. This finding that more calls completed correlates to mental health improvements further supports the hypothesis that coaching sessions may have a positive impact on an individual’s daily life. Change in depression and anxiety symptoms did not differ by sex, race, or education levels. Given that the sample size of 82 individuals was relatively small for this subgroup analysis, this finding should be further explored in future larger studies.

The results from this study indicate that participants had overall high satisfaction with the program. The majority of study participants reported the VC Health program improved their diet and eating habits, physical activity, cognitive ability, sleep habits, and stress levels. In addition, despite this being a year-long program, participants were engaged with the VC Health program throughout the study and averaged 16.5 coaching sessions over the 12-month period with an average of 12.9 min per coaching session.

A number of prospective clinical studies have been previously conducted to measure the impact of digital solutions on chronic disease prevention and management [37-40]; however, there have been a limited number of studies and solutions focused on older adults. In this study, we observed high compliance and engagement with study-related activities (eg, completion of RBANS tests, Web-based assessments) and high engagement and satisfaction with the digital technology–based VC Health program for a population of older at-risk adults with SCD. This suggests that older adults may also enjoy and benefit from new technology-based health solutions, such as the VC Health program, and can successfully participate in remote studies.

### Limitations

This study had a number of limitations, some of which have been previously discussed in the publication detailing this study’s methods and design [19]. First, given that this was a pre-post study design and there was no control arm, we were unable to compare the changes in cognitive ability and mental health experienced by individuals using the VC Health program with individuals who did not use the program. Therefore, we are unable to fully attribute the observed changes in cognitive ability and mental health to the VC Health program. However, previously conducted observational and longitudinal studies have shown that over a course of a 52-week period, older community-dwelling adults experienced a decline in cognitive ability (as measured by RBANS) over time [32,41]. This suggests that study participants’ cognitive ability may have declined over time in the absence of the VC Health program. Even if participants in the VC Health program just maintained cognitive ability over the course of the 52-week period, this would still be considered a better outcome than what would be seen in the absence of an intervention.

As a pilot study, the sample size was relatively small, primarily female, white, and well-educated. Although we did stratify results based on sex, race, and education levels in our analyses to explore differences in subgroups, sample sizes for each subpopulation were fairly small; therefore, conclusions based on stratified results are limited.

### Conclusions

In this pilot study, older adults at risk of developing AD experienced statistically significant improvements in their cognitive function and mental health after participating in the VC Health program. These results serve as initial evidence for the feasibility and effectiveness of this fully digital multidomain lifestyle change program (consisting of health coaching, diet, exercise, cognitive training, and social engagement components) to delay or prevent cognitive impairment in older adults at risk for developing AD. This initial evidence can be used to inform future longitudinal randomized controlled studies measuring the impact of the VC Health program on the prevention or delay of cognitive impairment.

## Abbreviations

AD: Alzheimer disease
BMI: body mass index
FINGER: Finnish Geriatric Intervention Study to Prevent Cognitive Impairment and Disability
GAD-7: Generalized Anxiety Disorder-7 survey
ITT: intention-to-treat
IQR: interquartile range
PHQ-9: Patient Health Questionnaire-9
RBANS: Repeatable Battery for the Assessment of Neuropsychological Status
SCD-9: Subjective Cognitive Decline questionnaire
VC Health: Virtual Cognitive Health
